# Image Encryption Algorithm Based on an Improved Tent Map and Dynamic DNA Coding

**DOI:** 10.3390/e27080796

**Published:** 2025-07-26

**Authors:** Wei Zhou, Xianwei Li, Zhenghua Xin

**Affiliations:** 1 School of Information Engineering, Suzhou University, Suzhou 234000, China; weizhou@ahszu.edu.cn (W.Z.);; 2 School of Computer and Information Engineering, Bengbu University, Bengbu 233000, China

**Keywords:** image encryption, chaotic map, Zigzag transform, pseudo-wavelet transform, DNA coding, chaotic performance evaluation, security analysis

## Abstract

As multimedia technologies evolve, digital images have become increasingly prevalent across various fields, highlighting an urgent demand for robust image privacy and security mechanisms. However, existing image encryption algorithms (IEAs) still face limitations in balancing strong security, real-time performance, and computational efficiency. Therefore, we proposes a new IEA that integrates an improved chaotic map (Tent map), an improved Zigzag transform, and dynamic DNA coding. Firstly, a pseudo-wavelet transform (PWT) is applied to plain images to produce four sub-images $I_{1}$, $I_{2}$, $I_{3}$, and $I_{4}$. Secondly, the improved Zigzag transform and its three variants are used to rearrange the sub-image $I_{1}$, and then the scrambled sub-image is diffused using XOR operation. Thirdly, an inverse pseudo-wavelet transform (IPWT) is employed on the four sub-images to reconstruct the image, and then the reconstructed image is encoded into a DNA sequence utilizing dynamic DNA encoding. Finally, the DNA sequence is scrambled and diffused employing DNA-level index scrambling and dynamic DNA operations. The experimental results and performance evaluations, including chaotic performance evaluation and comprehensive security analysis, demonstrate that our IEA achieves high key sensitivity, low correlation, excellent entropy, and strong resistance to common attacks. This highlights its potential for deployment in real-time, high-security image cryptosystems, especially in fields such as medical image security and social media privacy.

## 1. Introduction

The extensive progress in digital communication and multimedia applications has made the protection of image data increasingly important [1,2]. Images carrying sensitive data are frequently transmitted over insecure channels, such as the internet. Due to its large size and high redundancy, traditional encryption methods designed for text may not be efficient or suitable for image data [3]. Consequently, specialized IEAs have been developed to guarantee data confidentiality, integrity, and secure transmission [4,5]. These methods are widely used in medical imaging, military surveillance, cloud storage, and social media. However, existing encryption algorithms still have problems of weak security and low encryption efficiency. Therefore, there is an urgent need to design an exceptionally efficient and secure IEA.

Chaotic maps have the ability to produce highly unpredictable sequences, rendering them effective tools for disrupting the original structure of images. Because of their inherent sensitivity to initial conditions, unpredictability, and complex dynamic behavior, chaotic maps have garnered increasing attention and are anticipated to have a significant impact on modern image cryptosystems [6,7]. Naskar et al. introduced a robust IEA using a Tent map and cellular automata. This algorithm uses a Tent map and generates a dynamic key stream to encrypt image blocks, which enhances the overall security of the IEA [8]. Wang et al. developed a new 2D chaotic system using a Sine map to enhance performance. However, this algorithm requires multiple iterations, which is time-consuming [9]. Ghebleh et al. proposed an efficient IEA using chained skew Tent maps, in which the image is partitioned into multiple parts for encryption. This approach effectively disrupts the image data and improves the security [10]. Sneha et al. developed an IEA using the Arnold map, Tent map, and Walsh–Hadamard transform. The Walsh–Hadamard transform is first applied to the image to spread the pixel values, thereby enhancing the diffusion effect. Then, the Arnold and Tent maps are used to permute the image, resulting in a desirable permuted effect [11]. Mondal et al. integrated the skew Tent map with cellular automata as a key generator. Three pseudo-random numbers are generated for initializing cellular automata and the diffuse process [12]. However, the Tent map used in the above algorithm faces challenges such as discontinuity of chaotic intervals and the narrow chaotic range. To address these problems, we propose an improved Tent map that demonstrates continuous chaotic intervals and a broad chaotic range.

Permutation involves rearranging the pixel locations within an image, thereby disrupting its original structure and patterns [13], which makes the image difficult to recognize. Permutation is essential in image cryptosystems because it obscures visual information, thereby enhancing security, unpredictability, and resistance to statistical analysis. Wang et al. provided an IEA using the Zigzag transform and dynamic row scrambling. This algorithm uses index sequences produced by the two designed 1D chaotic maps to scramble plain images. The Zigzag transform is applied to the lower and upper triangular sections of the permuted image to further improve the permutation effect [14]. Hua et al. introduced a novel IEA that integrates an enhanced Zigzag transform with a value-differencing transform, which strengthens the algorithm’s security. The enhanced Zigzag transform uses chaotic sequences to generate a random scanning order, which enables more efficient pixel rearrangement [15]. Wang et al. designed an IEA that first scrambles the image using an extended Zigzag confusion scheme. Then, RNA operations are applied to the scrambled image, where the RNA encoding and decoding processes are realized using chaotic sequences [16]. Wang et al. segment the plain image into many blocks, and different Zigzag transforms are randomly selected for each block by using a cascaded chaotic map, thereby improving the randomness of the encrypted image [17]. Although researchers have provided many improved versions of the Zigzag transform, the scrambled image still exhibits a strong correlation in the horizontal direction. To address this issue, this paper designs an improved Zigzag transform and its three variants through bidirectional scanning and cross methods.

Diffusion disperses the pixel values throughout the entire image, making it visually hard to recognize and preventing decryption through statistical or other analytical methods [18,19]. Teng et al. applied DNA diffusion to the facial region identified through face recognition, enhancing the diffusion of the image data, thereby significantly strengthening the overall encryption robustness [20]. Zheng et al. developed an IEA that utilizes the Sine and Logistic maps. The image undergoes permutation using the Zigzag transform, followed by DNA addition and XOR operations for diffusion [21]. Nezhad et al. cross-reconstructed the bit planes of images to make the distribution of image data more even. To strengthen the encryption effect, DNA subtraction, addition, and XOR operations can be dynamically selected using an operation control sequence [22]. Lai et al. employed an integer wavelet transform and DNA coding for medical images. Then, the produced DNA sequence is shuffled using specialized DNA cubes to improve the security [23]. Anisha et al. combine DNA coding with an 8D hyperchaotic system to encrypt patient image data. The plain image is subjected to block scrambling, followed by dynamic DNA coding and XOR operations, where the coding and operations processes are controlled by a chaotic sequence [24]. However, using DNA coding alone as the diffusion method cannot provide sufficiently desirable diffusion effect. Therefore, this paper designs two new DNA operation rules and combines them with pixel-level diffusion method to enhance the diffusion effect.

In addition to the design of novel image encryption schemes, recent studies have also focused on analyzing their vulnerabilities through cryptanalysis. Chen et al. demonstrated that a DNA-based encryption scheme for DICOM images fails to withstand chosen-plaintext attacks [25]. Feng et al. demonstrated that the 2D Logistic-adjusted-Sine-map-based scheme suffers from weak key sensitivity, improper key stream generation, and a flawed permutation process, allowing chosen-plaintext attacks to fully recover the original image without access to secret keys [26]. Feng et al. conducted a detailed cryptanalysis of an image encryption scheme based on Feistel network and dynamic DNA encoding. They identify weaknesses in the secret key structure and encryption procedure and propose a targeted chosen-plaintext attack capable of fully recovering the plain image without secret keys [27]. Therefore, our proposed algorithm enhances resistance to plaintext attacks by employing a redesigned chaotic map with a broader chaotic range, an improved Zigzag transform and its three variants, and dynamically DNA operations.

Despite significant advancements in image encryption algorithms, several limitations remain unresolved. Many existing schemes still suffer from weak diffusion mechanisms, insufficient permutation strength, and limited chaotic behavior, which compromises their robustness against statistical and differential attacks. In particular, conventional chaotic maps often demonstrate narrow chaotic intervals and low key sensitivity, while standard Zigzag-based permutation methods leave strong directional correlations. Similarly, DNA-based diffusion techniques, though promising, are typically constrained by a small set of fixed operation rules, reducing unpredictability. These challenges highlight a pressing need for more adaptive and secure encryption strategies.

Motivated by these limitations, we introduce a comprehensive IEA that integrates an improved chaotic map, enhanced Zigzag permutation techniques, and dynamic DNA operations. The proposed approach aims to generate highly unpredictable encryption sequences, thoroughly disrupt spatial pixel correlations, and enrich diffusion behavior. Through the joint use of a redesigned Tent map, a set of four Zigzag variants, and novel compound DNA operation rules, the method ensures strong resistance to various types of attacks while maintaining encryption efficiency.

The contributions of our work include the following: (1) We design an improved Tent map by cascading the traditional Tent map and exponential function. Dynamics analyses demonstrate that it offers excellent randomness and complex chaotic behaviors. (2) To eliminate the limitations of the traditional Zigzag transform, we design an improved Zigzag transform and its three variants to enhance the scrambling effect. (3) To enrich the diversity of DNA operation rules, we design two novel DNA operations by compounding DNA addition and subtraction operations. (4) A two-layer IEA is proposed by combining the improved Tent map, improved Zigzag transform, and dynamic DNA coding, which can provide high-level security and efficiency. Through these innovations, our scheme achieves strong resistance against various plaintext and statistical attacks while maintaining computational efficiency.

The structure of this paper is as follows. Section 2 introduces the improved Tent map and its dynamics analyses. Section 3 presents the improved Zigzag transforms and our newly designed DNA operations. Section 4 describes the proposed encryption and decryption algorithms. Section 5 shows the experimental results and the security analyses. The conclusions are shown in Section 6.

## 2. Designed Chaotic Map

This section focuses on the construction and evaluation of a novel chaotic map aimed at enhancing the randomness and security of image cryptosystems. We begin by reviewing the traditional Tent map and identifying its limitations. Then, we introduce an improved Tent map designed to overcome these drawbacks. Comprehensive performance analyses, including sequence distribution, sensitivity, bifurcation behavior, the Lyapunov exponent, Shannon entropy, and NIST tests, were conducted to validate the chaotic properties and suitability of the improved map for secure encryption applications.

### 2.1. Traditional Tent Map

In image cryptosystems, the traditional Tent map [28] is a commonly used chaos because of its simple mathematical expression, which is defined as Equation (1).

(1) $$x_{n+1}=\left\{\begin{array}{cc} 2\alpha x_{n}, & x_{n}<0.5\\ 2\alpha (1-x_{n}), & x_{n}\geq 0.5 \end{array}\right.,$$

where *x* and α represent the state variable and control parameter, respectively. However, it has some drawbacks, such as a narrow chaotic range and uneven sequence distribution, limiting its application in high-security image cryptosystems.

### 2.2. Improved Tent Map

To overcome the drawbacks of the traditional Tent map, an improved Tent map is designed by combining it with exponential function and modulo operation. Equation (2) shows its mathematical expression.

(2) $$x_{n+1}=\left\{\begin{array}{cc} 2\mu (1-e^{2x_{n}})\;\;\mathrm{mod}\;\;1, & x_{n}<0.5\\ 2\mu (1-e^{2(1-x_{n})})\;\;\mathrm{mod}\;\;1, & x_{n}\geq 0.5 \end{array}\right.,$$

where μ∈(0,+∞) refers to the control parameter and x∈(0,1) represents the state variable.

### 2.3. Performance Analyses

#### 2.3.1. Sequence Distribution Diagram

**Figure 1:**
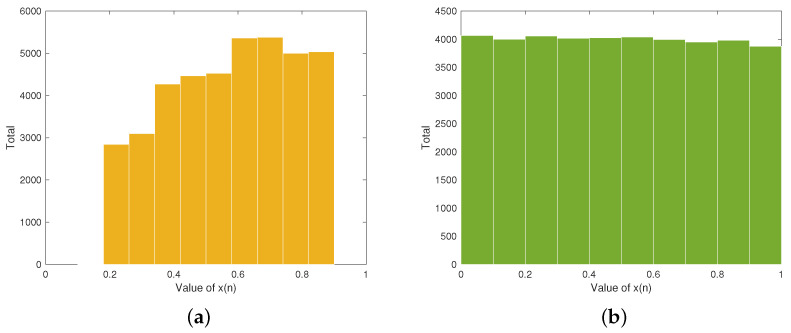
Sequence distribution diagrams: (**a**) traditional Tent map (α = 0.9 and x(0) = 0.1); (**b**) improved Tent map (μ = 50 and x(0) = 0.5).

The sequence distribution diagram provides a clear visualization of how chaotic sequences are spread across different intervals. Figure 1a illustrates the sequence distribution of the traditional Tent map. Obviously, the sequence distribution exhibits large fluctuation, which means that the generated sequence is non-uniform. Figure 1b displays the sequence distribution of our proposed Tent map. We can see from Figure 1b that the distribution of iteration values in each interval is approximately equal, which indicates uniform sequence distribution.

#### 2.3.2. Sensitivity

**Figure 2:**
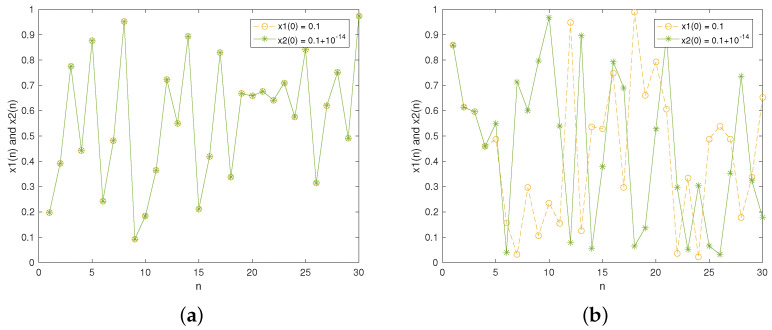
Sensitivity to initial value: (**a**) traditional Tent map (α=0.99); (**b**) improved Tent map (μ=50).

We slightly changed the initial values and then observed the resulting difference in the generated sequences. Figure 2 presents the results of the two chaotic maps where they are iterated 30 times. In Figure 2a, it is apparent that the two sequences completely overlap in the initial 30 iterations, indicating poor sensitivity. Figure 2b displays the experimental result of the improved Tent map. As can be seen, the two sequences diverge from the 5th iteration, meaning the designed improved Tent map exhibits strong sensitivity to changes in the initial value.

**Figure 3:**
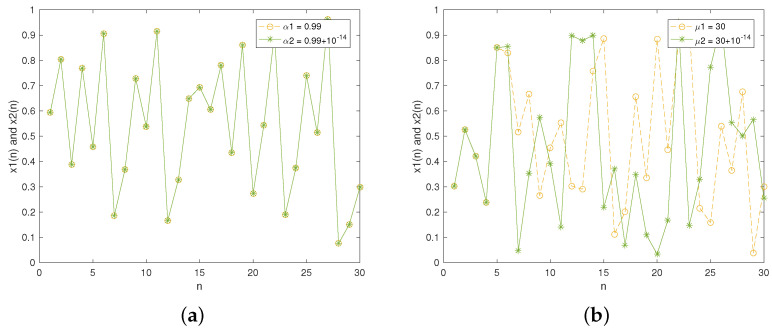
Sensitivity to control parameter: (**a**) traditional Tent map (x(0)=0.3); (**b**) improved Tent map (x(0)=0.1).

Figure 3 depicts the sensitivity to control parameters. For the traditional Tent map, Figure 3a shows the poor sensitivity to the control parameter, i.e., the two lines never diverge. However, for the improved Tent map, Figure 3b indicates the sequences begin to diverge from the 6th iteration, demonstrating its high sensitivity to changes in the control parameter.

#### 2.3.3. Bifurcation Diagram

**Figure 4:**
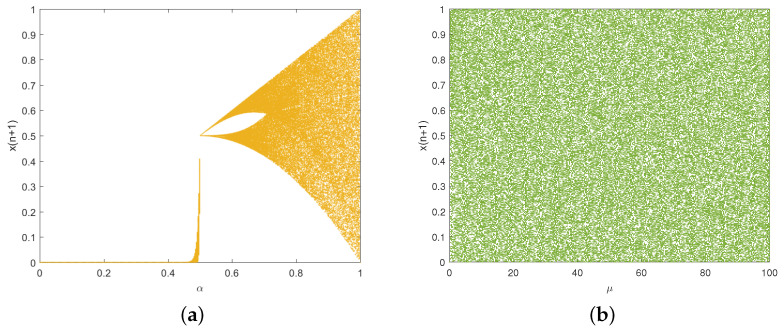
Bifurcation diagrams: (**a**) traditional Tent map; (**b**) improved Tent map.

The bifurcation diagram illustrates the qualitative behavior in a dynamic system as the control parameter varies. Figure 4 depicts the bifurcation diagrams of the traditional and improved Tent maps. For the two chaotic maps, the initial value $x_{0}$ was set to 0.5, then each map was iterated 3000 times. Figure 4a reveals that the traditional Tent map shows a limited chaotic range. Figure 4b indicates that our designed Tent map exhibits chaotic behavior almost across the full range of control parameter. This implies that the improved Tent map offers a broader chaotic range and greater unpredictability compared to the traditional one, which enhances its security in image cryptosystems.

#### 2.3.4. Lyapunov Exponent

The Lyapunov exponent (LE) quantifies how two nearby trajectories in a dynamical system diverge or converge over time [29]. It can be used to measure the sensitivity of a system to initial conditions. A dynamical system is considered in chaos when LE>0. Equation (3) gives the computing method of LE for a 1D chaotic map.

(3) $$LE=\lim_{n\to \infty }\frac{1}{n}\sum_{i=0}^{n-1}\ln|f^{\prime }\left(x_{i}\right)|,$$

where $f^{\prime }\left(x_{i}\right)$ represents the derivative of $f(x_{i})$ at the *i*th iteration.

**Figure 5:**
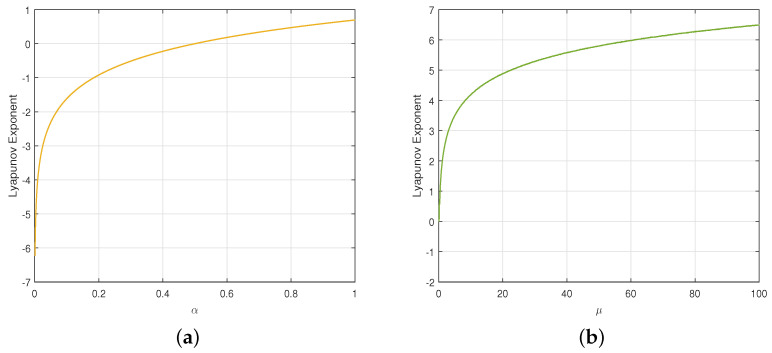
Lyapunov exponents: (**a**) traditional Tent map; (**b**) improved Tent map.

Figure 5 depicts the LE values for the two chaotic maps. Figure 5a indicates that LE>0 when the control parameter α∈(0.5,1), and the LE value is very small within the parameter range. For the improved Tent map, Figure 5b indicates LE>0 when the control parameter μ>0.15. Moreover, the improved Tent map demonstrates a higher LE value, indicating it has better sensitivity to its initial conditions.

#### 2.3.5. Shannon Entropy

**Figure 6:**
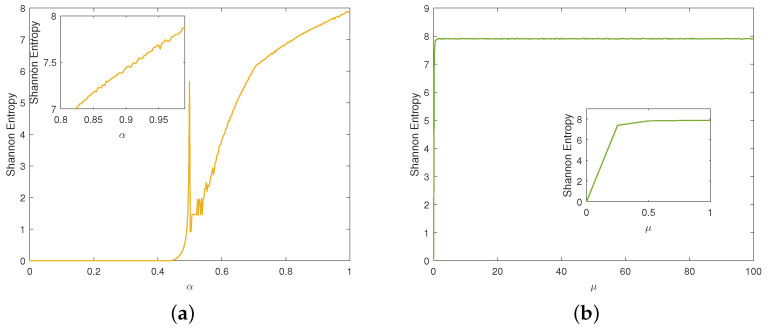
Shannon entropy: (**a**) traditional Tent map; (**b**) improved Tent map.

Shannon entropy quantifies the degree of uncertainty or unpredictability within a sequence. When applied to chaotic sequences, it helps quantify their randomness, information content, and suitability for applications such as encryption [30]. The computing method is shown in Equation (4).

(4) $$H\left(X\right)=-\sum_{i=1}^{n}p\left(x_{i}\right)\log_{2}p\left(x_{i}\right),$$

where $p(x_{i})$ represents the probability of the symbol $x_{i}$ occurring. A well-behaved chaotic sequence will yield an entropy value approaching the maximum 8. We conducted experiments to analyze the Shannon entropy of the two chaotic maps. For the traditional Tent map, Figure 6a illustrates that its entropy value approaches 8 only when α is close to 1. Figure 6b depicts that the improved Tent map consistently maintains an entropy value near its peak throughout almost the entire parameter range, demonstrating that it exhibits better randomness.

#### 2.3.6. NIST Test

The NIST test [31] is a statistical method used to examine the unpredictability of a sequence of values, ensuring it satisfies the necessary requirements for high-security application scenarios. A sequence passes the NIST test if its *p*-value > 0.01. The results of 15 sub-tests for the improved Tent map are displayed in Table 1. It is evident that the chaotic sequence successfully passes all sub-tests, confirming its excellent randomness. Through the above analyses of chaos performance, we can learn that the designed improved Tent map can be equipped on high-security image cryptosystems.

## 3. Designed Permutation and Diffusion Schemes

To enhance the overall encryption performance, this section introduces a well-structured permutation and diffusion scheme. The design is motivated by the need to effectively eliminate the spatial correlation between pixels and strengthen resistance against various types of attacks. Specifically, we improve the traditional Zigzag transform, which is widely used for permutation but suffers from directional limitations. In addition, to increase the diversity of DNA operation rules, we design two novel compound DNA operations by integrating DNA addition and subtraction. The combination of these techniques ensures both high security and computational efficiency.

### 3.1. Traditional Zigzag Transform

**Figure 7:**
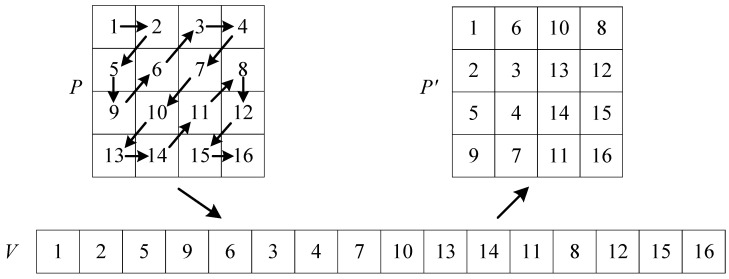
Scanning process of the traditional Zigzag transform.

In image encryption, the Zigzag transform is used as a permutation technique to disrupt spatial correlation of pixels and increase resistance against statistical and differential attacks. As depicted in Figure 7, the matrix *P* is scanned in a “*Z*” pattern using the traditional Zigzag transform. The resulting elements are stored sequentially in *V*, which is then converted into a matrix $P^{\prime }$. However, the traditional Zigzag transform has a weak scrambling effect, which limits its effectiveness in high-security image cryptosystems.

### 3.2. Improved Zigzag Transform

The traditional Zigzag transform follows a predictable path, typically scanning elements in a fixed “*Z*” shape. While effective to some degree, such patterns fail to fully disrupt strong pixel dependencies, especially in the horizontal direction. To address this, we propose an improved Zigzag transform designed to introduce scanning diversity.

**Figure 8:**
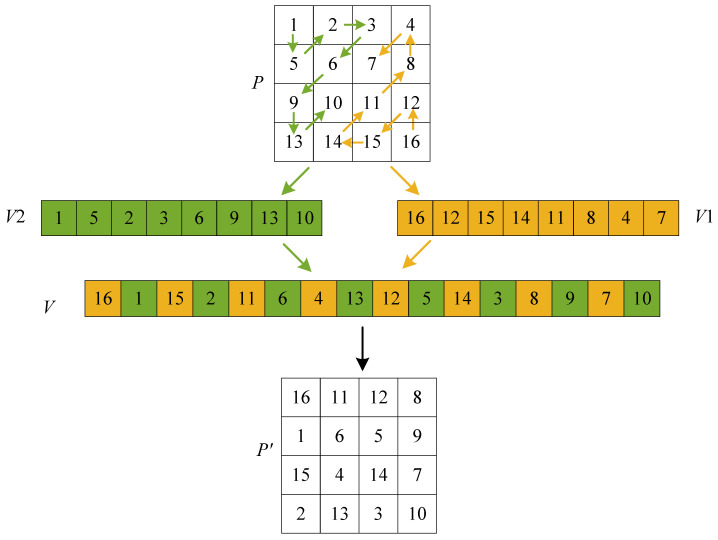
Scanning and cross processes of the improved Zigzag transform 1.

**Figure 9:**
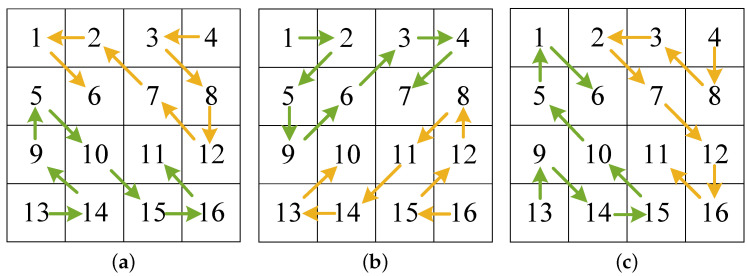
Improved Zigzag transforms: (**a**–**c**) improved Zigzag transform 2; (**b**) improved Zigzag transform 3; (**c**) improved Zigzag transform 4.

The traditional Zigzag transform scans the elements in a matrix in a sequential manner, which results in the high correlation between adjacent elements not being effectively reduced. To address this issue, this paper designs an improved Zigzag transform (improved Zigzag transform 1) and its three variants (improved Zigzag transforms 2, 3, and 4) through bidirectional scanning and odd-even cross methods. The designed improved Zigzag transform 1 is shown in Figure 8, and the other three variants are shown in Figure 9. The improved versions eliminate the limitation of the traditional Zigzag transform, thereby improving the scrambling effect. The specific implementation procedure for the improved Zigzag transform 1 is outlined below.

**Step** **1:** Reverse scanning

The pixels in the bottom-right part of a matrix *P* are traversed in a reverse manner until the $\left\lfloor mn/2\right\rfloor$th pixel is scanned. These pixels are then sequentially stored in a vector V1.

**Step** **2:** Forward scanning

The pixels in the upper-left part of *P* are traversed in a forward manner until the $\left\lceil mn/2\right\rceil$th pixel is scanned. These pixels are then sequentially stored in another vector V2.

**Step** **3:** Odd-even cross

The pixels in the odd and even positions in V1 are extracted, and then they are placed sequentially in the odd positions of *V*. Furthermore, the pixels in the odd and even positions in V2 are extracted, and then they are placed sequentially in the even positions of *V*.

**Step** **4:** Vector to matrix

*V* is then transformed into scrambled matrix $P^{\prime }$ of size m×n.

### 3.3. DNA Coding and DNA Operations

DNA coding is an innovative and biologically inspired technique used in image encryption. It translates pixel data into DNA nucleotide sequences and applies DNA-based rules to perform cryptographic operations. The complementary pairing relationships of the four bases in DNA are A-T and C-G. This complementary relationship also exists in electronic computer systems, i.e., 00-11 and 01-10. Consequently, each base can be represented by a two-bit binary. Table 2 illustrates 8 DNA coding rules that satisfy Watson–Crick base pairing principle [32]. We can use DNA encoding to map pixels to DNA sequences in image cryptosystems. For instance, 135 can be represented by a binary sequence “10000111”. Using encoding rule 1 in Table 2, the binary sequence “10000111” can be encoded into “GACT”. Subsequently, based on decoding rule 2 in Table 2, “GACT” can be decoded into “01001011”, i.e., 113.

DNA operations are core components in DNA-based image cryptosystems. They simulate biological operations on pixel data to change the value of pixels, enabling a secure and nonlinear transform of image content. Combined with chaotic maps and pixel permutation, they can provide high-level security. Common DNA operations include DNA addition and subtraction operations, as described in Table 3 and Table 4.

### 3.4. Designed Novel DNA Operation Rules

While basic DNA operations have shown promise in diffusion, their limited variety constrains the unpredictability of encryption. To expand the rules of DNA operations, we design two novel DNA compound operation rules based on the DNA addition and subtraction operations. Referring to coding rule 1 in Table 2, we describe the two newly designed DNA operation rules in detail below.

For the designed DNA addition and subtraction compound (ASC) operation, each base in the sequence <A, C, G, T> is increased by “00” when x=0, and we can obtain the result <A, C, G, T>. In addition, the sequence <A, C, G, T> is subtracted by “01” when x=1, and we can get the result <T, A, G, C>. After that, the sequence <A, C, G, T> is increased by “10” when x=2, and the result is <G, T, A, C>. Lastly, the sequence <A, C, G, T> is subtracted by “11” when x=3, resulting in <C, G, T, A>. The procedure for calculating the designed DNA ASC operation is shown in Equation (5).

(5) $$\begin{array}{c} f_{ASC}\left(x,<A,C,G,T>\right)=\left\{\begin{array}{c} <A+00,C+00,G+00,T+00>=<A,C,G,T>,x=0\\ <A-01,C-01,G-01,T-01>=<T,A,G,C>,x=1\\ <A+10,C+10,G+10,T+10>=<G,T,A,C>,x=2\\ <A-11,C-11,G-11,T-11>=<C,G,T,A>,x=3 \end{array}\right.. \end{array}$$

Similarly, for the designed DNA subtraction and addition compound (SAC) operation, the sequence <A, C, G, T> is subtracted by “00” when x=0, and we can obtain the result <A, C, G, T>. Furthermore, the sequence <A, C, G, T> is increased by “01” when x=1, and we can get the result <C, G, T, A>. Afterwards, the sequence <A, C, G, T> is subtracted by “10” when x=2, and the result is <G, T, A, C>. Finally, the sequence <A, C, G, T> is increased by “11” when x=3, resulting in <T, A, C, G>. The computation procedure for the designed DNA SAC operation is shown in Equation (6).

(6) $$\begin{array}{c} f_{SAC}\left(x,<A,C,G,T>\right)=\left\{\begin{array}{c} <A-00,C-00,G-00,T-00>=<A,C,G,T>,x=0\\ <A+01,C+01,G+01,T+01>=<C,G,T,A>,x=1\\ <A-10,C-10,G-10,T-10>=<G,T,A,C>,x=2\\ <A+11,C+11,G+11,T+11>=<T,A,G,C>,x=3 \end{array}\right.. \end{array}$$

Table 5 and Table 6 present the designed DNA ASC and SAC operations, where the two designed DNA compound operations are inverse of each other.

Compound rules based on chaotic control inputs introduce higher diffusion complexity and nonlinearity. Traditional DNA operations, though effective, are limited to a small, deterministic set of transformations. Our designed ASC and SAC operations expand the rules of DNA operations, increasing unpredictability and enhancing the avalanche effect. This makes them more resilient to statistical, brute-force, and differential attacks. Performance evaluation in Section 5 further confirms that the proposed operations outperform existing ones in terms of the NPCR, UACI, and entropy.

## 4. Proposed Encryption Algorithm

To ensure both strong security and high efficiency, we propose a novel IEA that integrates an improved Tent map, four variants of improved Zigzag transforms, and newly designed compound DNA operations. The improved Tent map provides continuous chaotic intervals, a broad chaotic range, and uniform sequence distribution. The Zigzag transform variants significantly enhance permutation complexity, while the compound DNA operations increase diffusion effectiveness.

### 4.1. Key Generation

The plain image is converted into a 256-bit binary hash value based on the SHA-256 algorithm. Next, the 256-bit binary is divided into groups of 8 bits, and each group is converted into its corresponding decimal form, resulting in 32 decimal hash values K={k1,k2,…,k32}. These values are then combined with three externally provided secret keys $\varepsilon_{1},\varepsilon_{2}$ and $\varepsilon_{3}$ to generate the initial values and control parameters of the improved Tent map. This hybrid method of key derivation enhances key sensitivity and expands the key space while preserving simplicity in hardware and software implementation. The key generation formula is provided in Equation (7).

(7) $$\left\{\begin{array}{c} u_{1}=\varepsilon_{1}+\frac{\;\;\;\mathrm{mod}\;\;\;(k_{1}+k_{32},256)}{10,000}\\ u_{2}=\varepsilon_{2}+\frac{\;\;\;\mathrm{mod}\;\;\;(k_{2}+k_{30}+k_{31},256)}{10,000}\\ u_{3}=\varepsilon_{3}+\frac{\;\;\;\mathrm{mod}\;\;\;(k_{3}+k_{28}+k_{29},256)}{10,000}\\ x_{0}^{\prime }=\frac{k_{4}⊕k_{6}⊕k_{8}⊕k_{10}⊕k_{12}⊕k_{14}⊕k_{16}⊕k_{18}}{256}\\ x_{0}^{\prime \prime }=\frac{k_{5}⊕k_{7}⊕k_{9}⊕k_{11}⊕k_{13}⊕k_{15}⊕k_{17}⊕k_{19}}{256}\\ x_{0}^{\prime \prime \prime }=\frac{\left(k_{20}⊕k_{21}⊕k_{22}⊕k_{23}\right)+\left(k_{24}⊕k_{25}⊕k_{26}⊕k_{27}\right)}{1024} \end{array}\right.\;,$$

where ⊕ and mod(·) represent XOR and modulo operations, respectively.

### 4.2. Encryption Algorithm

**Figure 10:**
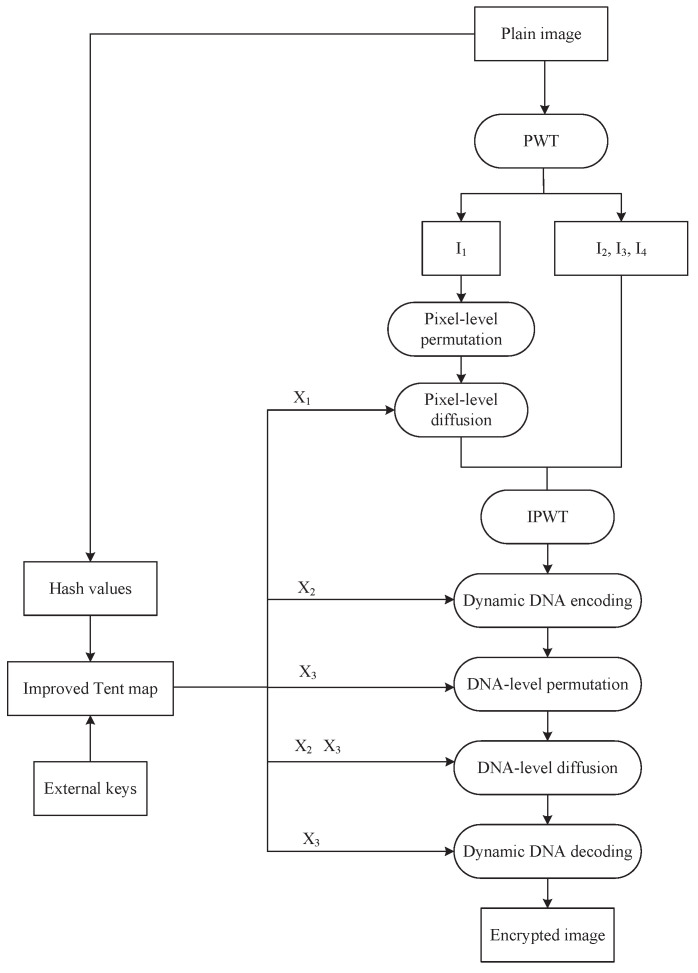
Flowchart of encryption process.

The encryption procedure is constructed in two layers—pixel-level and DNA-level encryption—to maximize entropy and enhance obfuscation. The proposed IEA begins with the application of the pseudo-wavelet transform (PWT) [33]. Subsequently, pixel-level permutation and diffusion are performed to obtain a semi-encrypted image. Following that, we apply DNA-level permutation and diffusion to generate the encrypted image. The encryption process is depicted in Figure 10.

**Step** **1:** Chaotic sequence generation

We generate the initial values and control parameters $x_{0}^{\prime },x_{0}^{\prime \prime },x_{0}^{\prime \prime \prime },u_{1},u_{2}$, and $u_{3}$ according to Equation (7). And then, three chaotic sequences $X_{1}$, $X_{2}$, and $X_{3}$ are produced using the improved Tent map. The length of $X_{1}$ is mn/4, while the lengths of $X_{2}$ and $X_{3}$ are both 4mn.

**Step** **2:** PWT

Image *P* is decomposed using PWT to obtain four sub-images $I_{1}$$I_{2}$, $I_{3}$, and $I_{4}$, each of size m×n/4.

**Step** **3:** Pixel-level permutation

We randomly select one of the four improved Zigzag transforms using a chaotic sequence. The selection process involves calculating the mean value *x* of *P*, defined by Equation (8). Then, we convert *x* into $x^{\prime }$ by Equation (9).

(8) $$\begin{array}{c} x=\frac{1}{m\times n}\sum_{i=1}^{m}\sum_{j=1}^{n}P(i,j), \end{array}$$

(9) $$\begin{array}{c} x^{\prime }=\;\;\mathrm{mod}\;\;\left(x,4\right)+1, \end{array}$$

where $x^{\prime }\in \left\{1,2,3,4\right\}$. Next, we permute the sub-image $I_{1}$ using the improved Zigzag transform to generate $P_{1}$, which has a size of m×n/4.

**Step** **4:** Pixel-level diffusion

We apply Equation (10) to convert $X_{1}$ into a pixel sequence *C*. According to Equation (11), the diffused sub-image *D* is obtained by XORing $P_{1}$ with *C*.

(10) $$\begin{array}{c} C\left(i\right)=mod\left(floor(X_{1}\left(i\right)\times 10^{15}),256\right),\;i=1,2,\ldots ,\frac{mn}{4}. \end{array}$$

(11) $$\begin{array}{c} D\left(i\right)=D\left(i-1\right)⊕C\left(i\right)⊕P_{1}\left(i\right). \end{array}$$

Subsequently, an inverse pseudo-wavelet transform (IPWT) is applied to *D*, $I_{2}$, $I_{3}$, and $I_{4}$ to generate image $P_{2}$, which has a size of m×n.

**Step** **5:** Dynamic DNA encoding

We generate the encoding rule $R_{enc}$ according to Equation (12).

(12) $$R_{enc}\left(i\right)=mod(floor(10^{3}\times |X_{2}\left(i\right)|),8)+1,i=1,2,\ldots ,4mn,$$

where |·| denotes the absolute value function, and floor(·) denotes the round-down function. We perform dynamic DNA encoding on $P_{2}$ to produce a DNA sequence $P_{3}$ of length 4mn.

**Step** **6:** DNA-level permutation

$X_{3}$ is sorted to generate an index sequence $I_{idx}$. Then, $P_{3}$ is permuted according to Equation (13) to generate a permuted DNA sequence $P_{4}$.

(13) $$P_{4}\left(i\right)=P_{3}\left(I_{idx}\left(i\right)\right),i=1,\;2,\ldots ,4mn.$$

**Step** **7:** DNA-level diffusion

We convert the chaotic sequence $X_{2}$ into a DNA sequence $C_{DNA}$ according to Equation (14). The operation process utilizes four DNA operation rules: DNA addition and subtraction operations specified in Table 3 and Table 4, DNA ASC operation, and DNA SAC operation from Table 5 and Table 6. The inclusion of DNA-level operations significantly improves the diffusion strength and further increases the unpredictability of the encrypted image. The dynamic DNA operation rule $R_{oper}$ is generated from the chaotic sequence $X_{3}$, provided by Equation (15).

(14) $$\begin{array}{c} C_{DNA}\left(i\right)=mod(floor(|X_{2}\left(i\right)|\times 10^{4}),\;4),i=1,\;2,\ldots ,4mn. \end{array}$$

(15) $$\begin{array}{c} R_{oper}\left(i\right)=mod(floor(|X_{3}\left(i\right)|\times 10^{4}),\;4)+1,i=1,\;2,\ldots ,4mn. \end{array}$$

Dynamic DNA operations are performed using Equation (16) and $P_{5}$ is the DNA sequence after operations.

(16) $$P_{5}\left(i\right)=\left\{\begin{array}{c} f_{ADD}\left(P_{4}\left(i\right),C_{DNA}\left(i\right)\right),R_{oper}\left(i\right)=1\\ f_{SUB}\left(P_{4}\left(i\right),C_{DNA}\left(i\right)\right),R_{oper}\left(i\right)=2\\ f_{ASC}\left(P_{4}\left(i\right),C_{DNA}\left(i\right)\right),R_{oper}\left(i\right)=3\\ f_{SAC}\left(P_{4}\left(i\right),C_{DNA}\left(i\right)\right),R_{oper}\left(i\right)=4 \end{array}\right..$$

**Step** **8:** Dynamic DNA decoding

We generate the decoding rule $R_{dec}$ according to Equation (17).

(17) $$R_{dec}\left(i\right)=mod(floor(10^{3}\times |X_{3}\left(i\right)|),8)+1,i=1,2,\ldots ,4mn.$$

Then, dynamic DNA decoding is performed on $P_{5}$ to generate a binary sequence $P_{6}$ of length 4 mn. After that, we convert $P_{6}$ into a decimal sequence $P_{7}$ of length mn. Finally, $P_{7}$ is reshaped into an encrypted image $P^{\prime }$.

### 4.3. Decryption Algorithm

**Figure 11:**
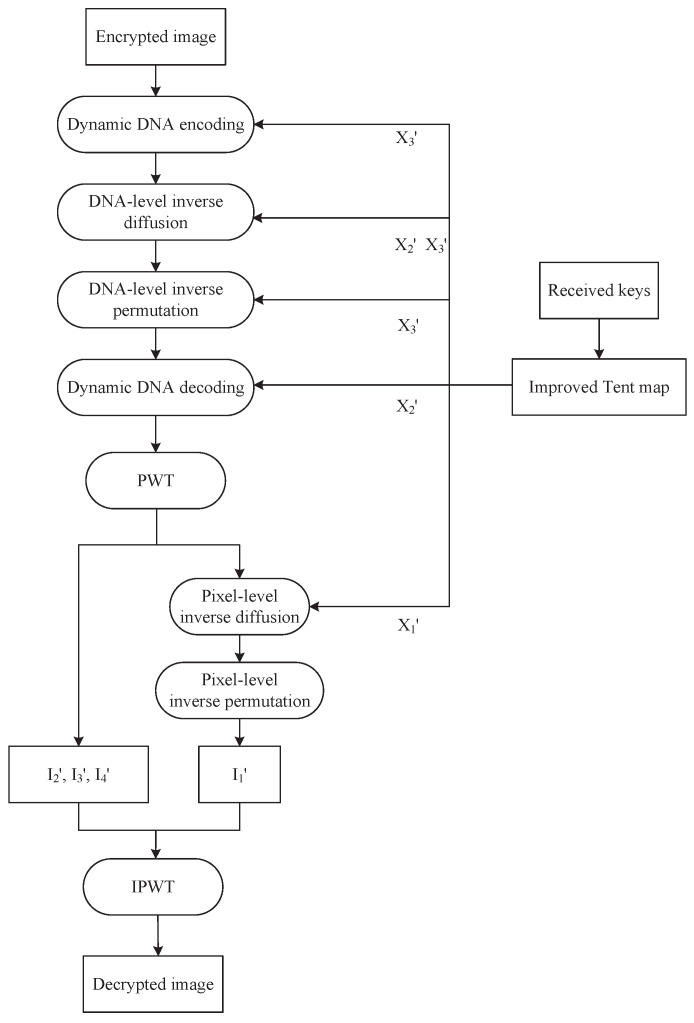
Flowchart of decryption process.

The decryption process is completely reversible, provided the keys and rules are correctly matched. This ensures both high security and lossless recovery of the original image. Figure 11 shows the flowchart of the decryption process. We first apply dynamic DNA coding, DNA-level inverse diffusion, and permutation to obtain the semi-decrypted image. And then, pixel-level inverse diffusion and permutation are performed to generate the final decrypted image.

**Step** **1:** Chaotic sequence generation

We generate three chaotic sequences $X_{1}^{\prime },X_{2}^{\prime }$, and $X_{3}^{\prime }$ using the improved Tent map and the received keys $x_{0}^{\prime \prime },x_{0}^{\prime \prime \prime },x_{0}^{\prime \prime \prime \prime },u_{1}^{\prime },u_{2}^{\prime }$, and $u_{3}^{\prime }$, where the length of $X_{1}^{\prime }$ is mn/4, the lengths of $X_{2}^{\prime }$ and $X_{3}^{\prime }$ are both 4mn.

**Step** **2:** Dynamic DNA encoding

We reshape the encrypted image $P^{\prime }$ into a vector $P_{1}^{\prime }$ of length mn and then convert $P_{1}^{\prime }$ into a binary sequence $P_{2}^{\prime }$. The encoding rule $R_{\mathrm{enc}}^{\prime }$ is generated according to Equation (18).

(18) $$R_{enc}^{\prime }\left(i\right)=mod\left(floor\left(10^{3}\times \left|X_{3}^{\prime }\left(i\right)\right|\right),8\right)+1,\;i=1,2,\ldots ,4mn.$$

Afterwards, dynamic DNA encoding is performed on $P_{2}^{\prime }$ to generate a DNA sequence $P_{3}^{\prime }$.

**Step** **3:** DNA-level inverse diffusion

$X_{2}^{\prime }$ is converted into a chaotic DNA sequence $C_{\mathrm{DNA}}^{\prime }$ according to Equation (19).

(19) $$C_{\mathrm{DNA}}^{\prime }\left(i\right)=mod\left(floor\left(|X_{2}^{\prime }\left(i\right)|\times 10^{4}\right),4\right),\;i=1,2,\ldots ,4mn.$$

The dynamic DNA operation rule $R_{oper}^{\prime }$ is generated from $X_{3}^{\prime }$, as illustrated in Equation (20).

(20) $$R_{oper}^{\prime }\left(i\right)=mod(floor(|X_{3}^{\prime }\left(i\right)|\times 10^{4}),4)+1,i=1,2,\ldots ,4mn.$$

$P_{4}^{\prime }$ is the DNA sequence after inverse diffusion, and the inverse diffusion is performed using Equation (21).

(21) $$P_{4}^{\prime }\left(i\right)=\left\{\begin{array}{cc} f_{SUB}\left(P_{3}^{\prime }\left(i\right),C_{\mathrm{DNA}}^{\prime }\left(i\right)\right), & R_{\mathrm{oper}}^{\prime }\left(i\right)=1\\ f_{ADD}\left(P_{3}^{\prime }\left(i\right),C_{\mathrm{DNA}}^{\prime }\left(i\right)\right), & R_{\mathrm{oper}}^{\prime }\left(i\right)=2\\ f_{ASC}\left(P_{3}^{\prime }\left(i\right),C_{\mathrm{DNA}}^{\prime }\left(i\right)\right), & R_{\mathrm{oper}}^{\prime }\left(i\right)=3\\ f_{SAC}\left(P_{3}^{\prime }\left(i\right),C_{\mathrm{DNA}}^{\prime }\left(i\right)\right), & R_{\mathrm{oper}}^{\prime }\left(i\right)=4 \end{array}\right..$$

**Step** **4:** DNA-level inverse permutation

We sort $X_{3}^{\prime }$ to generate an index sequence $I_{idx}^{\prime }$. Then, $P_{4}^{\prime }$ is scrambled using DNA-level inverse permutation in Equation (22), resulting in a DNA sequence $P_{5}^{\prime }$.

(22) $$P_{5}^{\prime }\left(I_{\mathrm{idx}}^{\prime }\left(i\right)\right)=P_{4}^{\prime }\left(i\right),\;i=1,2,\ldots ,4mn.$$

**Step** **5:** Dynamic DNA decoding

The decoding rule $R_{dec}^{\prime }$ is generated by Equation (23).

(23) $$R_{dec}^{\prime }\left(i\right)=mod(floor(10^{3}\times |X_{2}^{\prime }\left(i\right)|),8)+1,\;i=1,2,\ldots ,4mn.$$

We perform dynamic DNA decoding on sequence $P_{5}^{\prime }$ to generate a decoded sequence $P_{6}^{\prime }$ of length mn.

**Step** **6:** Pixel-level inverse diffusion

PWT is used to decompose $P_{6}^{\prime }$ into four sub-images: $D^{\prime }$, $I_{2}^{\prime }$, $I_{3}^{\prime }$, and $I_{4}^{\prime }$, each with size m×n/4. Then, we use Equation (24) to convert $X_{1}^{\prime }$ into a chaotic DNA sequence $C^{\prime }$.

(24) $$C^{\prime }\left(i\right)=mod\left(floor(X_{1}^{\prime }\left(i\right)\times 10^{15}),256\right),\;i=1,2,\ldots ,\frac{mn}{4}.$$

According to Equation (25), $P_{7}^{\prime }$ is obtained by XORing $D^{\prime }$ with sequence $C^{\prime }$.

(25) $$P_{7}^{\prime }\left(i\right)=D^{\prime }\left(i\right)⊕D^{\prime }\left(i-1\right)⊕C^{\prime }\left(i\right).$$

**Step** **7:** Pixel-level inverse permutation

One of the four improved Zigzag inverse transforms are randomly selected for the inverse permutation. This process involves calculating the mean value *y* of $P_{7}^{\prime }$, as shown in Equation (26). And then, we convert *y* into $y^{\prime }$ by using Equation (27).

(26) $$\begin{array}{c} y=\frac{1}{m\times n}\sum_{i=1}^{m}\sum_{j=1}^{n}P_{7}^{\prime }\left(i,j\right), \end{array}$$

(27) $$\begin{array}{c} y^{\prime }=\;\;\mathrm{mod}\;\;\left(y,4\right)+1, \end{array}$$

where $y^{\prime }\in \left\{1,2,3,4\right\}$. Then, we permute $P_{7}^{\prime }$ using the improved Zigzag inverse transform to generate $I_{1}^{\prime }$, which has a size of m×n/4.

**Step** **8:** IPWT

IPWT is applied to sub-images $I_{1}^{\prime }$, $I_{2}^{\prime }$, $I_{3}^{\prime }$, and $I_{4}^{\prime }$, resulting in the decrypted image *P*.

## 5. Experimental Results and Algorithm Analyses

### 5.1. Experimental Results

**Figure 12:**
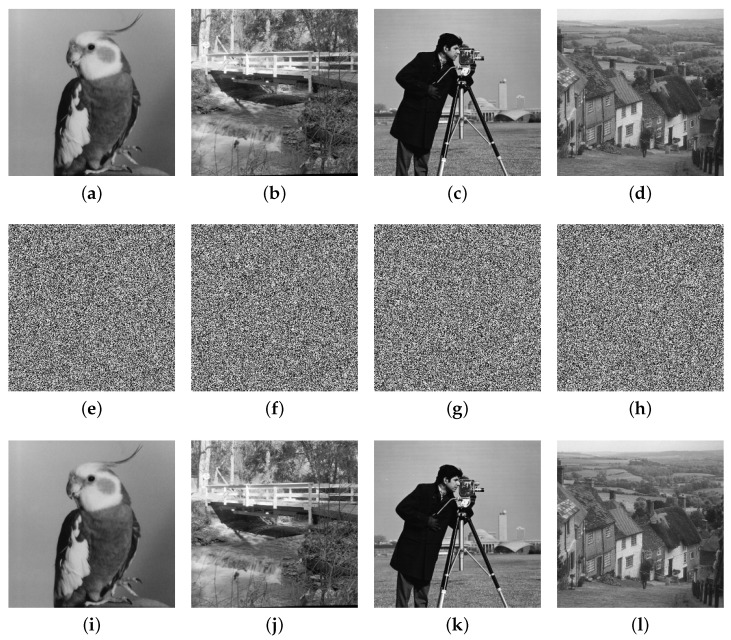
Experimental results: (**a**–**d**) Bird, Bridge, Camera, and Goldhill plain images; (**e**–**h**) encrypted images; (**i**–**l**) decrypted images.

We conducted our experiments in MATLAB R2022a on a computer equipped with an 11th Gen Intel(R) Core(TM) i5-11300H @ 3.10 GHz CPU and 16 GB RAM. The used Bird, Bridge, Camera, and Goldhill (256×256) gray images are from https://links.uwaterloo.ca/Repository.html (accessed on 20 April 2025), and we present the experimental results in Figure 12. It is evident that the encrypted images cannot reveal any meaningful content through visual inspection, and they can be correctly decrypted when the correct keys are applied.

### 5.2. Key Space

Larger key spaces provide more security because they make brute-force attacks more computationally expensive and time-consuming. For our proposed IEA, the key is composed of the initial conditions of the designed chaos, i.e., $x_{0}^{\prime },x_{0}^{\prime \prime },x_{0}^{\prime \prime \prime },u_{1},u_{2}$, and $u_{3}$, with value ranges $x_{0}^{\prime }\in \left(0,1\right),x_{0}^{\prime \prime }\in \left(0,1\right),x_{0}^{\prime \prime \prime }\in \left(0,1\right),u_{1}\in \left(0,+\infty \right),u_{2}\in \left(0,+\infty \right)$, and $u_{3}\in \left(0,+\infty \right)$. With a computer precision of $10^{-15}$, the key space of our IEA is $10^{15\times 6}=10^{90}\approx 2^{300}$, which far exceeds the minimal threshold of $2^{128}$ typically mandated for cryptographic schemes [34,35]. Therefore, the algorithm we proposed is difficult to crack by brute force attacks.

We analyzed the robustness of the key generation mechanism. Although the theoretical key space of our IEA is approximately $10^{84}$, floating-point operations are inherently subject to rounding errors and the limitations of finite machine precision. To mitigate this issue, the proposed algorithm employs double-precision arithmetic, ensuring at least 15 decimal digits of accuracy. Moreover, all chaotic iterations are performed under fixed high-precision software-level control to minimize key space degradation and enhance security.

### 5.3. Key Sensitivity

**Figure 13:**
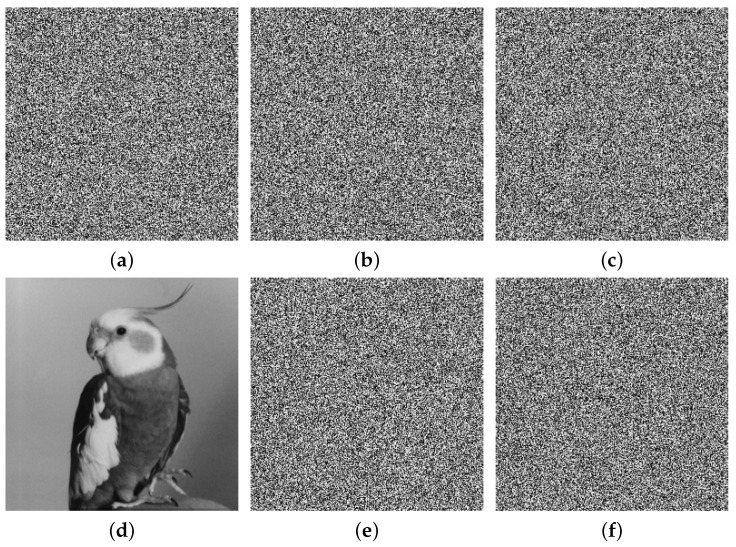
Key sensitivity analyses: (**a**–**c**) encrypted images with SK0, SK1, and SK2, respectively; (**d**–**f**) decrypted images with SK0, SK1, and SK2, respectively.

The keys $x_{0}^{\prime }$, $x_{0}^{\prime \prime }$, $x_{0}^{\prime \prime \prime }$, $\mu_{1}$, $\mu_{2}$, and $\mu_{3}$ are represented by SK0. We perturb $\mu_{1}$ by adding $10^{-14}$, resulting in SK1. Similarly, modifying $x_{0}^{\prime }$ by adding $10^{-14}$ results in SK2. Figure 13a–c shows the encrypted Bird images using SK0, SK1, and SK2, respectively. Figure 13d–f displays the decrypted images of Figure 13a using SK0, SK1, and SK2, respectively. The number of pixels change rate (NPCR) [36] is used to describe the difference between two images. We can see from Table 7 that a small modification in any key leads to significantly different encrypted images. Thus, our IEA is exceptionally sensitive to modifications in the key, which is a desirable feature for high-security image cryptosystems.

### 5.4. Histogram

**Figure 14:**
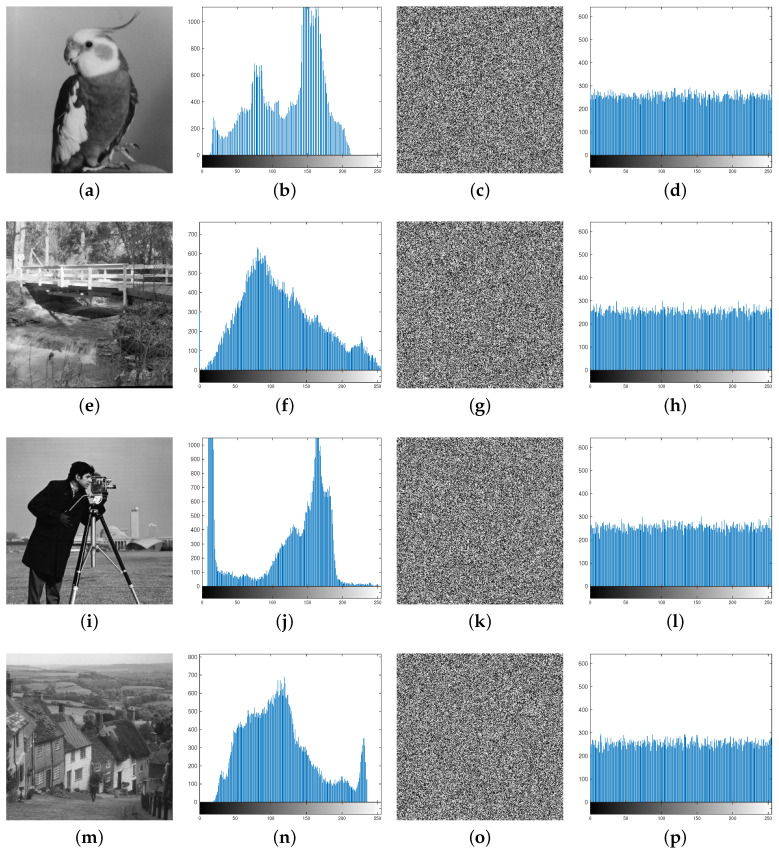
Histograms: the second column shows the histograms of the plain images; the fourth column displays the histograms of the encrypted images.

In the context of image encryption, a histogram represents the frequency distribution of pixel intensities in an image. It is a powerful statistical tool for analyzing the performance and security of an encryption algorithm. A flatter histogram in the encrypted image indicates its enhanced ability to resist statistical analysis attacks. In Figure 14, we can see that the histograms in the fourth column are even, suggesting that our IEA can effectively resist statistical analysis attacks.

### 5.5. Correlation

**Figure 15:**
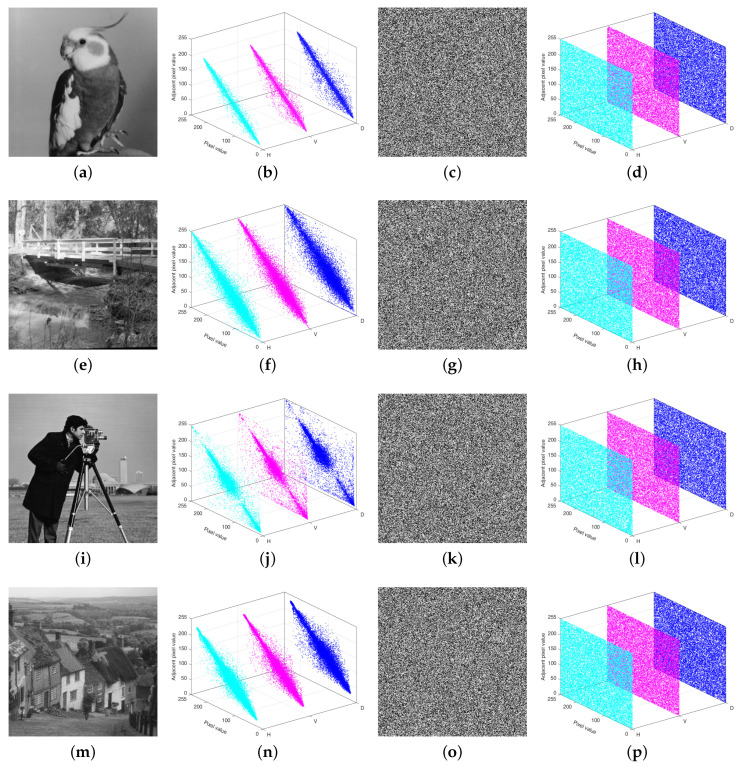
Correlation distribution diagrams: the second column shows the correlation distribution diagrams of the plain images; the fourth column displays the correlation distribution diagrams of the encrypted images.

Hackers can analyze the correlation between adjacent pixels and conduct statistical analysis attacks, so reducing the correlation is necessary. The correlation can be quantitatively analyzed using a correlation coefficient [37], and its formula is provided in Equation (28).

(28) $$r=\frac{\sum_{i=1}^{M}\left(x_{i}-E\left(x\right)\right)\left(y_{i}-E\left(y\right)\right)}{\sqrt{\sum_{i=1}^{M}{\left(x_{i}-E\left(x\right)\right)}^{2}\sum_{i=1}^{M}{\left(y_{i}-E\left(y\right)\right)}^{2}}},\;$$

where E(·) denotes the mathematical expectation. In Table 8, the correlation coefficients of the encrypted images are close to 0. The correlation distribution diagram can analyze the correlation from a qualitative perspective, as shown in Figure 15. Table 8 and Figure 15 indicate that our proposed IEA demonstrates superior statistical performance compared to algorithms reported in Refs. [38,39,40].

### 5.6. Information Entropy

Information entropy [41] can reflect information uncertainty; specifically, the more significant the uncertainty, the greater the information entropy. Equation (29) presents the formula for information entropy.

(29) $$H\left(X\right)=-\sum_{i=1}^{n}p\left(x_{i}\right)\log_{2}\left(p\left(x_{i}\right)\right),$$

where $p(x_{i})$ is the probability of $x_{i}$ appearing. Table 9 presents the entropy values for images encrypted when using different IEAs. As we can see, the encrypted images produced by our algorithm make the entropy values closer to 8. This suggests that our IEA exhibits strong resistance to entropy-based attacks.

### 5.7. Differential Attacks

The NPCR and Unified Average Changing Intensity (UACI) [46,47] are used to assess the sensitivity of encrypted images to slight variations in the plain image. NPCR reflects the percentage of pixels that differ in value, whereas UACI measures the overall magnitude of the differences in pixel values. Their computing methods are defined in Equations (30) and (31).

(30) $$\begin{array}{c} NPCR=\frac{\sum_{i=1}^{m}\sum_{j=1}^{n}D(i,j)}{m\times n}, \end{array}$$

(31) $$\begin{array}{c} UACI=\frac{\sum_{i=1}^{m}\sum_{j=1}^{n}\left|C_{1}\left(i,j\right)-C_{2}\left(i,j\right)\right|}{255\times m\times n}, \end{array}$$

where $C_{1}$ and $C_{2}$ denote two encrypted images that differ by only a single pixel in their plain versions. D(i,j)=0 when the pixel values at corresponding positions are the same, whereas D(i,j)=1 when the pixel values are different. Table 10 demonstrates that our algorithm can make NPCR and UACI values approach their theoretical values 99.6094 and 33.463507. Compared to other IEAs in Refs. [48,49,50], our algorithm closely approximates the ideal values, highlighting its robust resilience against differential attacks.

### 5.8. Robustness

**Figure 16:**
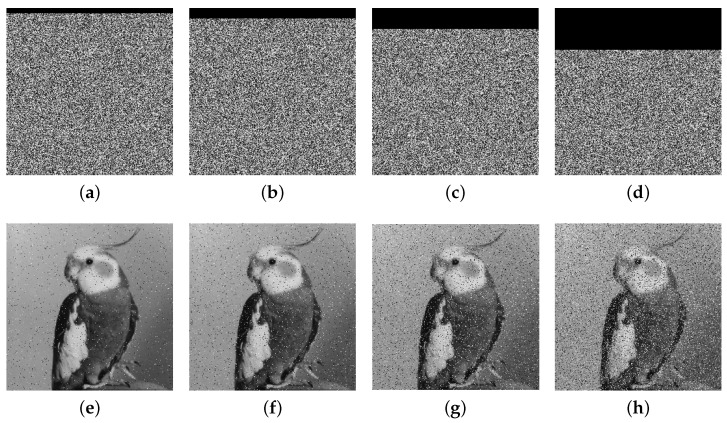
Cropping attack analyses: (**a**–**d**) cropped encrypted images; (**e**–**h**) decrypted images.

**Figure 17:**
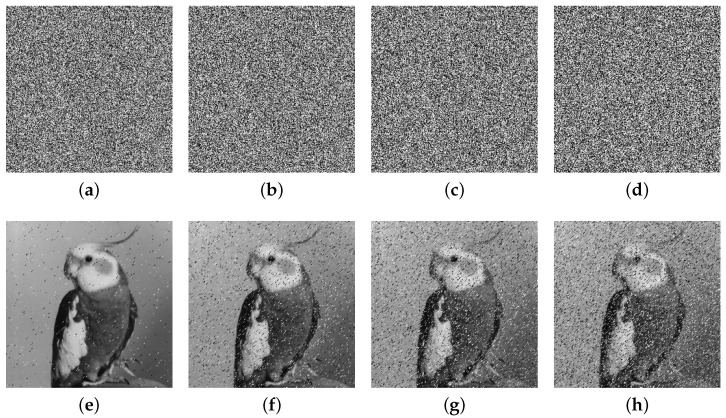
Noise attack analyses: (**a**–**d**) noise-added encrypted images; (**e**–**h**) decrypted images.

We conducted experiments by sequentially cropping the Bird encrypted images with 1/32, 1/16, 1/8, and 1/4 proportions, followed by decryption of the cropped encrypted images. The results depicted in the second row in Figure 16 illustrate the effect of anti-cropping attacks. Additionally, we introduce salt-and-pepper noise to the encrypted images, as shown in Figure 17. The results demonstrate that even under cropping and noise distortions, the algorithm retains a significant ability to recover features of the original image. Thus, our developed algorithm exhibits robust resistance against cropping and noise attacks.

### 5.9. Execution Time

We executed our algorithm on a 256×256 Lena image 20 times, and the average time taken per step is presented in Table 11. We can see that the encryption process requires 0.150985 s. Table 12 presents the encryption times of our algorithm and the algorithms reported in Refs. [51,52,53,54,55]. Obviously, our algorithm achieves faster encryption speeds, which makes it more suitable in real-time application scenarios.

## 6. Conclusions

With the increasing demand for privacy protection in image data transmission and storage, the design of secure and efficient image encryption algorithms remains a significant challenge. Many existing image encryption schemes struggle to achieve a satisfactory balance among security strength, randomness, computational efficiency, and real-time performance. To address these issues, we propose a novel IEA that integrates an improved Tent map, four variants of improved Zigzag transforms, and newly designed compound DNA operations.

The improved Tent map achieves outstanding chaotic performance by providing continuous chaotic intervals, broad chaotic range, and uniform sequence distribution. The improved Zigzag transform and its three variants provide flexible and effective scrambling strategies, significantly enhancing permutation complexity. Additionally, the incorporation of dynamic DNA encoding and two newly designed compound DNA operation rules increases diffusion capability.

The extensive experimental results and security analyses demonstrate that the proposed IEA achieves excellent performance in terms of key sensitivity, information entropy, pixel correlation, resistance to differential attacks, and robustness under noise and cropping. The encryption time for a 256×256 gray image is approximately 0.1509 s, confirming the algorithm’s suitability for real-time, high-security applications, such as medical image protection and secure communication in social platforms.

Despite these strengths, the current scheme is primarily validated on gray images. In future work, we plan to extend the algorithm to color images and video encryption, further optimize the execution time on resource-limited devices, and explore its integration with compression and watermarking technologies to support hybrid image security frameworks.

## Figures and Tables

**Table 1:** NIST test: Please, confirm table’s alignment. results of the improved Tent map.

No.	Sub-Test	*p*-Value	Pass/Fail
1	Frequency	0.259319	✓
2	Approximate entropy	0.188924	✓
3	Block frequency	0.260430	✓
4	Runs	0.721295	✓
5	Overlapping template	0.608952	✓
6	Linear complexity	0.805624	✓
7	Non-overlapping template	0.759281	✓
8	FFT	0.071990	✓
9	Rank	0.019179	✓
10	Serial	0.404234	✓
11	Longest run	0.492331	✓
12	Cumulative sums	0.499166	✓
13	Universal statistical	0.209323	✓
14	Serial test	0.695586	✓
15	Random excursions	0.636530	✓

**Table 2:** DNA encoding/decoding rules.

Rule	1	2	3	4	5	6	7	8
A	00	00	01	01	10	10	11	11
C	01	10	00	11	00	11	01	10
T	11	11	10	10	01	01	00	00
G	10	01	11	00	11	00	10	01

**Table 3:** DNA addition operation.

+	A	C	G	T
A	A	C	G	T
C	C	G	T	A
G	G	T	A	C
T	T	A	C	G

**Table 4:** DNA subtraction operation.

−	A	C	G	T
A	A	T	G	C
C	C	A	T	G
G	G	C	A	T
T	T	G	C	A

**Table 5:** Designed DNA ASC operation.

ASC	A	C	G	T
A	A	T	G	C
C	C	A	T	G
G	G	C	A	T
T	T	G	C	A

**Table 6:** Designed DNA SAC operation.

SAC	A	C	G	T
A	A	C	G	T
C	C	G	T	A
G	G	T	A	C
T	T	A	C	G

**Table 7:** NPCR values between any two encrypted images.

Encrypted Image	Key Set	NPCR (%)		
		Figure 13a	Figure 13b	Figure 13c
Figure 13a	SK0	0	99.6490	99.5956
Figure 13b	SK1	99.6490	0	99.6002
Figure 13c	SK2	99.5956	99.6002	0

**Table 8:** Comparison of correlation coefficients for different algorithms.

Algorithm	Horizontal	Vertical	Diagonal
Lena	0.9703	0.9453	0.9208
Ours	0.0001	0.0026	0.0017
Ref. [38]	0.0032	0.0009	0.0067
Ref. [39]	−0.0148	0.0106	0.0134
Ref. [40]	−0.0024	−0.0054	−0.0129

**Table 9:** Comparison of information entropy.

Images	Ours	Ref. [42]	Ref. [43]	Ref. [44]	Ref. [45]
Lena (256×256)	7.9978	7.9972	7.9968	7.9973	7.9954
Cameraman (256×256)	7.9976	7.9974	7.9971	7.9972	7.9949
Peppers (256×256)	7.9977	7.9969	7.9970	7.9974	7.9948
House (256×256)	7.9978	7.9972	7.9972	-	-
Lena (512×512)	7.9995	-	-	-	-
Peppers (512×512)	7.9994	-	-	-	-
Baboon (512×512)	7.9995	-	-	-	-
Peppers (1024×1024)	7.9999	-	-	-	-
Male (1024×1024)	7.9999	-	-	-	-

**Table 10:** Comparisons of NPCR and UACI for different images and algorithms.

Image	Ours		Ref. [48]		Ref. [49]		Ref. [50]	
	NPCR (%)	UACI (%)	NPCR (%)	UACI (%)	NPCR (%)	UACI (%)	NPCR (%)	UACI (%)
Lena	99.6078	33.4678	99.6106	33.4781	99.6086	33.4320	99.5892	33.4358
Peppers	99.6081	33.4668	99.6074	33.404	99.6265	33.5192	99.5815	33.4946
Baboon	99.6122	33.4716	99.6054	33.4531	99.6254	33.4583	99.6171	33.5964
Boat	99.6065	33.4782	99.604	33.4355	-	-	99.6233	33.4304
Lake	99.6078	33.4796	99.6111	33.5233	-	-	99.5595	33.4418

**Table 11:** Running time of each step (in seconds).

Step	Time	Percentage
Chaotic sequence generation	0.045039	29.83%
PWT	0.001727	1.14%
Pixel-level permutation	0.005143	3.41%
Pixel-level diffusion	0.034137	22.61%
Dynamic quaternary DNA encoding	0.009081	6.01%
DNA-level permutation	0.013189	8.74%
DNA-level diffusion	0.008685	5.75%
Dynamic quaternary DNA decoding	0.009052	6.00%
Other	0.024932	16.51%
Total	0.150985	100%

**Table 12:** Encryption time comparison (in seconds) under different input scales.

Algorithm	Input Scale		
	256×256	512×512	1024×1024
Ours	0.1509	0.5748	2.3148
Ref. [51]	0.9654	2.8263	-
Ref. [52]	-	0.8970	-
Ref. [53]	0.3381	-	-
Ref. [54]	0.4409	-	-
Ref. [55]	0.6000	-	-

## Data Availability

Data will be made available on reasonable request.
